# Posttranscriptional Regulation of Splicing Factor SRSF1 and Its Role in Cancer Cell Biology

**DOI:** 10.1155/2015/287048

**Published:** 2015-07-26

**Authors:** Vânia Gonçalves, Peter Jordan

**Affiliations:** ^1^Department of Human Genetics, National Health Institute Dr. Ricardo Jorge, Avenida Padre Cruz, 1649-016 Lisboa, Portugal; ^2^Biosystems and Integrative Sciences Institute (BioISI), Faculty of Sciences, University of Lisbon, 1749-016 Lisboa, Portugal

## Abstract

Over the past decade, alternative splicing has been progressively recognized as a major mechanism regulating gene expression patterns in different tissues and disease states through the generation of multiple mRNAs from the same gene transcript. This process requires the joining of selected exons or usage of different pairs of splice sites and is regulated by gene-specific combinations of RNA-binding proteins. One archetypical splicing regulator is SRSF1, for which we review the molecular mechanisms and posttranscriptional modifications involved in its life cycle. These include alternative splicing of SRSF1 itself, regulatory protein phosphorylation events, and the role of nuclear versus cytoplasmic SRSF1 localization. In addition, we resume current knowledge on deregulated SRSF1 expression in tumors and describe SRSF1-regulated alternative transcripts with functional consequences for cancer cell biology at different stages of tumor development.

## 1. Introduction

The expression of a gene is initiated by its transcription into a precursor messenger RNA (mRNA), which is then further processed and spliced into a mature mRNA. Splicing is regulated through the interaction between RNA-binding proteins (RBPs) and their cognate splicing regulatory sequence elements (SREs) in the mRNA. This is especially important for alternative splicing where multiple mRNAs can be generated from the same pre-mRNA through the joining of selected exons or usage of different pairs of splice sites [1].

The number of genes encoding RBPs in the human genome is currently estimated to be around 860 [2, 3], far below the number of around 200 000 transcripts that can be produced from the roughly 21 000 human protein-coding genes. Therefore, a key principle in splicing regulation is that the interaction of RNA-binding proteins with SREs is not a one-to-one relationship: each SRE motif can be recognized by multiple alternative RBPs and most splicing factors can recognize two or more SRE motifs. This is particularly relevant for alternative splicing events, the regulation of which involves a complex network of competing protein-RNA interactions so that individual exons can be controlled by multiple factors [4, 5]. For example, multiple RNA-binding proteins with similar splicing regulatory activities might bind the same motif and this functional redundancy creates robustness in a splicing decision. Also, some factors compete with or displace another factor with opposite activity and confer functional antagonism. These overlapping binding specificities allow regulatory plasticity, which underlies tissue-specific splicing patterns, subtle fine-tuning of splice variant levels, and regulatory relationships between splicing regulators and upstream signaling pathways.

Among the RBPs, the major classes of splicing factors that control splice site recognition are the families of Serine/Arginine-rich (SR) proteins and heterogeneous nuclear ribonucleoproteins (hnRNPs). These proteins act by selecting splice sites for recognition by the spliceosome through binding to intronic or exonic splice enhancer and silencer elements and promoting or destabilizing protein interactions with spliceosome components. One of the best studied factors is SRSF1, formerly known as ASF or SF2 [6]. SRSF1 is a prototypical splicing factor mostly recruited to SREs classified as exonic splicing enhancers (ESEs). SRSF1 recognizes degenerate purine-rich sequence motifs [7, 8] and its binding promotes recognition of both constitutive and alternative exons during spliceosomal assembly. The current knowledge about its regulation will be the focus of this review. The described principles of regulation also apply to many other SR proteins and RBPs.

## 2. Posttranscriptional Regulation of SRSF1 mRNA

The SRSF1 gene is essential for normal embryonic development that is constitutively expressed and tightly regulated at the posttranscriptional level. In particular, SRSF1 recognizes SREs in its own transcripts, leading to alternative splicing, with some transcript forms being degraded by nonsense-mediated mRNA decay (NMD). In case of SRSF1, alternative splicing occurs in the 3′ untranslated region following excision of an additional intron and thus introduction of a new exon-exon junction. In consequence, the original stop codon is recognized as premature and the transcript targeted for NMD [9]. This mechanism is highly conserved and shared by other SR proteins. It serves both as a negative feedback loop, in which increased SR protein levels promote an increase in unproductive splice variants of their own transcripts, and as a target for regulation, for example, depending on the ERK1/2-mediated phosphorylation status of the splicing regulator Sam68 [10].

In addition, other posttranscriptional mechanisms of SRSF1 autoregulation were described such as nuclear retention of alternative SRSF1 transcript variants or regulation of the translational efficiency of its transcripts [11, 12]. Furthermore, miRNAs targeting SRSF1 translation have begun to be identified, including miR-28, miR-505, miR-10a, and miR-10b [13, 14]. Thus, SRSF1 transcript levels are fine-tuned by various posttranscriptional mechanisms but the quantitative contribution of each step and their orchestration in response to different cellular stimuli remains undetermined.

## 3. SRSF1 Regulation by Protein Phosphorylation

Following translation of SRSF1 transcripts into protein, constitutive phosphorylation steps occur. First, the predominantly cytoplasmic SR-specific protein kinases (SRPKs) phosphorylate part of the C-terminal Arg-Ser-rich (RS) domain, which contains 20 serine residues. SRPK1 was shown to phosphorylate the proximal first 12 residues [15] and this promotes nuclear import through interaction of phospho-SRSF1 with the import factor transportin-SR2 [16] and subsequent localization into nuclear speckles [17, 18].

**Figure 1:**
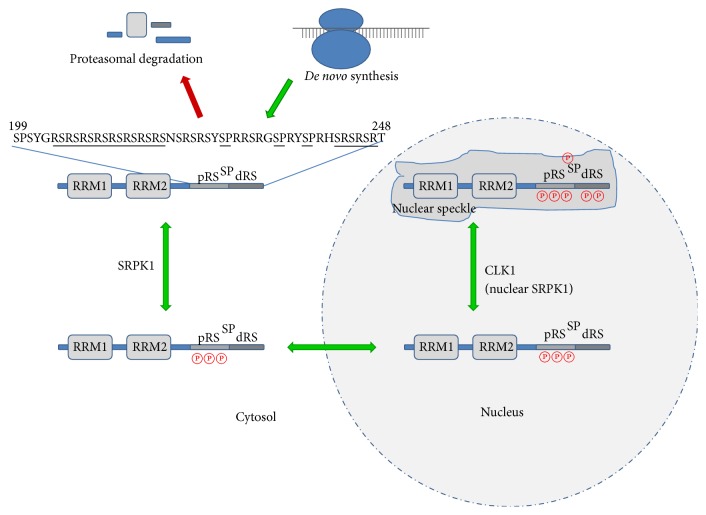
Life cycle and posttranslational modifications of the SRSF1 protein. Following its* de novo* synthesis at ribosomes, the cytoplasmic SRSF1 protein is constitutively phosphorylated by the cytosolic protein kinase SRPK at serine residues in the proximal RS domain (pRS). This first phosphorylation step is required for nuclear import of SRSF1, followed by a second phosphorylation step at the distal RS domain (dRS), including several serine-proline (SP) motifs. This step is usually catalyzed by the nuclear protein kinase CLK1 but can also be performed by SRPK if induced to translocate into the nucleus. Nuclear translocation is further modulated through methylation of the three arginine residues R93, R97, and R109 located between the two RNA recognition motif (RRM) domains (not shown). SRSF1 with a completely phosphorylated RS domain accumulates in nuclear speckles from where it is recruited to the spliceosome. SRSF1 dephosphorylation induces its nuclear to cytoplasmic translocation and lack of phosphorylation by SRPK in the cytosol leads to its proteolytic degradation.

Once in the nucleus the Cdc2-like kinases (CLKs) phosphorylate the remaining serine residues in the distal RS domain which leads to dispersed nuclear localization of SRSF1 and is required for its function in splicing [19–21] through cotranscriptional association with RNA polymerase II (pol2). Upon transcription inhibition SRSF1 is translocated from the nucleoplasm back to nuclear speckles [22] (see Figure 1 for a graphic summary).

Recently, it was found that SRPK1 can also shuttle into the nucleus [23–26] where then SRPK1 and CLK1 display similar activities toward Arg-Ser repeats in the distal RS domain, suggesting that these kinases no longer operate in a strict linear manner along the RS domain. Instead, CLKs appear to recognize preferentially the three Ser-Pro dipeptides in the RS domain, the phosphorylation of which has been proposed to change the conformation of the RS domain and regulate SRSF1 contact sites required in the spliceosome [27]. Nuclear translocation of SRPK1 can be induced, for example, by stress conditions, and involves disruption of its binding to a cytoplasmic Hsp70/Hsp90 complex [28].

Besides these phosphorylation events considered to be constitutive, other protein kinases have been reported to regulate SRSF1 through phosphorylation.

Protein kinase A (PKA) can phosphorylate SRSF1 on serine 119* in vitro* and modulate its activity as a splicing factor [29, 30]. This phosphorylation occurs in the so-called pseudo-RNA recognition motif (RRM) and was described to change the RNA-binding properties of SRSF1 and reduce its capacity to activate splicing.

Human DNA topoisomerase I (topo I) has also been described to phosphorylate SRSF1 [31, 32], most likely in the RS domain. This phosphorylation promotes the binding of SRSF1 to cognate ESEs during alternative splicing events [33]. The DNA damage signal poly-ADP ribose forms a complex with SRSF1, and this promotes Topo I to switch from its protein kinase to DNA relaxation activity [34]. A further connection between SRSF1 and Topo I is their role in preventing R-loop formation, stable mRNA:DNA hybrids that can form following transcription [35, 36].

Another kinase reported to phosphorylate SRSF1* in vitro* is AKT [37, 38], which also targets serine residues in the RS domain, leading to altered splicing decisions. A subsequent study reported that AKT1 interacts with and promotes SRPK1 and SRPK2 autophosphorylation and their subsequent translocation into the nucleus [26] with simultaneous formation of a phosphatase containing complex to downregulate AKT activity [39]. It remains to be established whether AKT phosphorylates SRSF1 directly or whether the described activity of immunoprecipitated AKT to phosphorylate SRSF1 originates from associated SRPKs.

The serine/threonine kinase NEK2 is also a splicing factor kinase that colocalizes with SRSF1 in nuclear speckles. It interacts with and phosphorylates SRSF1, affecting the splicing activity of SRSF1 in a SRPK1-independent manner [40].

## 4. SRSF1 Regulation through Nuclear-Cytoplasmic Distribution

Ample experimental evidence showed that SRSF1 is a shuttling protein that localizes to both the nucleus and the cytoplasm, depending on the phosphorylation state of its RS domain [37, 41]. Furthermore, experimental blocking of SRPK (by either depleting its expression level or inhibiting its kinase activity) revealed that the cytoplasmic pool of SRSF1 increased, identifying phosphorylation as a major factor for SRSF1 nuclear translocation [42, 43]. The contribution of nuclear phosphatase activity to cytoplasmic export of SRSF1 has not been directly demonstrated* in vivo* but protein phosphatase 1 can dephosphorylate the proximal RS domain of SRSF1* in vitro* or in permeabilized cell nuclei [44–46].

One physiological condition modulating SRSF1 localization is stress response, when general splicing is inhibited but specific alternative splicing events continue to occur [47]. For example, replicative senescence or induced stress stimuli of the vascular endothelium result in preferential cytoplasmic localization of SRSF1 and the underlying mechanism was postulated to involve nuclear import of SRPK1 and consequently lack of constitutive cytosolic SRSF1 phosphorylation [48]. In contrast, hyperphosphorylation of SRSF1 was observed during the DNA damage response and caused altered subnuclear distribution and changes in alternative splicing pattern of target genes [49].

Another posttranslational modification involved in SRSF1 localization is the methylation of three arginine residues (R93, R97, and R109) located in a region between the two RRM domains [50]. Lack of methylation in a triple-Ala mutant turned SRSF1 predominantly cytoplasmic, whereas a triple-Lys substitution maintaining the positive charge localized to nuclear speckles, as the wild-type protein. How the respective protein arginine methyltransferases (PRMTs) are regulated and contribute to the nuclear-cytoplasmic transitions of SRSF1 is poorly understood.

Once in the nucleus, the long noncoding RNA MALAT1 (metastasis-associated lung adenocarcinoma transcript 1) interacts with SRSF1, which is important for the recruitment of other SR proteins into nuclear speckles [51].

## 5. Functional Consequences of Nuclear versus Cytoplasmic SRSF1 Concentrations

Relative concentrations of antagonizing or competing SFs are important determinants in alternative splicing regulation. For example, SRSF1 generally displays a stimulatory role in splicing when bound to exons and its function in alternative splicing* in vitro* can be antagonized by the activity of hnRNP A proteins in a concentration-dependent manner.* In vivo*, competition between SFs can originate from the relative ratios of such antagonists expressed in different tissues or developmental stages, creating tissue or stage-specific patterns of splicing. In addition, the dynamic regulation of subcellular SF localization allows cells to modulate the effective nuclear concentration of a given SF and alter the pattern of expressed splicing variants in response to external stimuli. For example, the subnuclear distribution of SRSF1 changes during the DNA damage response following hyperphosphorylation and results in a shift in the alternative splicing pattern of target genes that control cell survival [49]. Also, drug-induced disruption of nuclear speckles with concomitant release of SRSF1 into the nucleoplasm induced changes in alternative splicing events [52]. And endothelial senescence is associated with a scattered distribution of SRSF1 throughout the cytoplasm. This leads to the expression of alternative isoforms of target genes such as endoglin (ENG), vascular endothelial growth factor A (VEGFA), tissue factor (T3), or lamin A (LMNA) that integrate into a common molecular senescence program [48]. Vice versa, epithelial cells treated with insulin-like growth factor-1 (IGF-1) displayed nuclear translocation of SRSF1, which was dependent on SPRK1/2 activity.

## 6. Regulation of SRSF1 by Cytosolic Protein Degradation

A specific decrease in SRSF1 protein levels was observed in SRPK1-depleted or SRPIN340-treated colorectal cells, without changes in the corresponding SRSF1 mRNA. This suggests that cytoplasmic SRSF1 localization leads to protein degradation. Indeed, the SRSF1 protein remained stable in such treated cells when incubated with inhibitors MG132 or lactacystin, indicating degradation by the proteasome [43]. It should be noted that studying SFs with proteasome inhibitors needs to be well controlled at the corresponding transcript level because the inhibitors are likely to affect other SFs or transcription factors in the cell. For example, the gene encoding SRSF3 (former SRp20) is a direct transcriptional target of *β*-catenin/TCF4 [53] so that inhibition of *β*-catenin degradation will increase expression of SRSF3, which in turn can promote unproductive alternative SRSF1 transcripts [54].

SRSF1 protein expression levels did also not correlate with mRNA expression levels following T cell stimulation. Immunoprecipitation studies showed increased ubiquitylation of SRSF1 in activated T cells and proteasomal but not lysosomal degradation was shown to be involved by blocking with specific inhibitors MG132 and bafilomycin, respectively. Interestingly, T cells from patients with SLE (systemic lupus erythematosus) showed increased ubiquitylation of SRSF1 when compared to those from healthy individuals [55].

Downregulation of SRSF1 protein level was further found to occur following inhibition of activity or siRNA-mediated depletion of GSK3*β* in U87 or U373 glioblastoma cells [56]. Similarly, GSK3*β* depletion in HT29 colorectal cancer cells led to a reduction in both SRSF1 and SRPK1 protein levels, suggesting an indirect effect of GSK3*β* on SRSF1 via SRPK1 [43].

It has been described that the RS domain, which is common to all SR proteins, is required for their proteolytic degradation by the proteasome [57] but further mechanistic details remain to be determined.

## 7. Impact of Posttranslational Modification on Other RNA-Related Functions of SRSF1

SRSF1 has been shown to facilitate the nuclear export of spliced mRNAs to which it is bound through its interaction with the TAP/NXF1 receptor [58]. Interestingly, this adaptor function implies partial dephosphorylation of its RS domain for cytoplasmic translocation [59], suggesting the phosphorylation status of SRSF1 serves to regulate nuclear export of some mRNPs.

The subsequent ribosomal translation of transcripts containing a SRSF1-targeted ESE is also stimulated, both* in vivo* and* in vitro* [60]. Thus, SRSF1-mediated alternative splicing, mRNA export, and translational efficiency of its target transcripts are coupled, and their number has been identified experimentally to be around 500 [61].

In addition, SRSF1 overexpression was found to increase the ratio between cap-dependent and internal ribosome entry site-dependent translation initiation [62], probably by suppressing the activity of 4E-BP, a competitive inhibitor of cap-dependent translation [63]. Probably related to these properties is the observation that SRSF1 enhances nonsense-mediated mRNA decay (NMD) [64] because SRSF1 overexpression can promote the pioneer round of translation required for NMD to occur [58].

Another class of RNA pol2 transcripts is miRNAs and SRSF1 overexpression in HeLa cells promoted the maturation step of miR-7 and other miRNAs. SRSF1 (and also other splicing factors) directly interacts with primary miR transcripts and promotes the Drosha cleavage step generating mature miRNA [65].

Another recently discovered function of SRSF1 is to enhance protein sumoylation [66]. SRSF1 associates with the SUMO E2 conjugating enzyme Ubc9 and enhances SUMO conjugation to RNA processing factors but further details on the regulation or consequences of this modification remain to be identified.

It should also be noted that SRSF1 was shown to be involved in chromatin organization and histone modifications such as H3K36me3, which are relevant for splicing decisions [67, 68].

## 8. Role of SRSF1 in Cancer Cell Biology and Tumorigenesis

Malignant changes in the cellular genome can either be tumor-initiating driver events or subsequent adaptations required for tumor cell progression. Such changes either alter the expression level of critical genes or their nucleotide sequence to generate gain- or loss-of-function mutant gene products. Although point mutations in core components of the spliceosome were recently discovered using whole-genome sequencing approaches [69], reports from various tumor types revealed that splicing factors mostly show increased expression levels [70–72]. Concerning SRSF1, overexpression was reported in tumors from colon, thyroid, small intestine, kidney, lung, liver, pancreas, and breast [73, 74]. In childhood acute lymphoblastic leukemia SRSF1 was further found to be upregulated together with protein arginine methyltransferase PRMT1 [75], which is involved in promoting SRSF1 nuclear localization [50].

The overexpression of SRSF1 in tumors has been related to several alternative mechanisms. First, in breast tumors and breast cancer cell lines amplification of the* SFRS1* gene at chromosomal location 17q23 was detected and the increased DNA copy number correlated with elevated SRSF1 mRNA levels [73].

Second, the* SRSF1* gene is a target of MYC, a potent oncogenic transcription factor overexpressed in many different tumor types that has pleiotropic effects on cancer cell biology [76]. MYC binds directly to the SRSF1 promoter and activates transcription. Both genes were found coexpressed in lung and breast carcinomas and MYC depletion downregulates SRSF1 expression in lung cancer cell lines [77].

Third, the above-mentioned negative feedback loop, in which SRSF1 promotes an increase in unproductive splice variants of its own transcripts, can be subverted in the presence of splicing regulator SAM68. Changes in expression or phosphorylation of SAM68 were found to promote the formation of full-length SRSF1 transcripts, thus leading to increased SRSF1 protein levels [10]. SAM68 phosphorylation depends on ERK/MAP kinase activity, which is frequently augmented in human tumors.

Together, this indicates that SRSF1 overexpression is in general the consequence of other preceding tumor-initiating genetic changes but contributes to further tumor progression.

Two apparently opposing consequences of SRSF1 overexpression on cancer cell biology have been described: the induction of oncogene-induced senescence and the malignant transformation of cells. On the one hand, SRSF1 overexpression leads to the formation of a nucleoplasmic complex with the ribosomal protein RPL5 and the E3-ubiquitin ligase MDM2, which normally ubiquitylates the p53 tumor-suppressor protein leading to its proteolytic degradation [78]. Complex formation inhibits MDM2 and thus p53 protein levels increase and trigger a cellular senescence response, which normally is part of a ribosomal stress pathway. Because the ability of SRSF1 overexpression to activate a tumor-suppressing senescence response is dependent on an intact p53 pathway, the identified SRSF1-overexpressing tumor types revealed characteristics of p53 inactivation [78].

On the other hand, SRSF1 can act as an oncogene since a twofold increase in expression can transform immortalized rodent fibroblasts [73, 79] and human mammary epithelial cells [73, 79]. In these models, SRSF1 overexpression promoted cell proliferation and antiapoptotic pathways, mainly reflecting the combined effects of several alternative splicing variants which were activated by the concentration-dependent changes in SRSF1 availability. Some of these specific variants have been characterized, as detailed below, but probably represent just the tip of the iceberg.

One group of identified target genes is formed by the apoptosis regulators BIN1, BCL2L11 (BIM), BCL-XL, ICAD, and MCL1, with SRSF1 overexpression in cancer cells promoting the formation of their respective antiapoptotic splice variants. Several target genes belong to the Bcl-2 family of proteins, which regulate whether the Bak and Bax proteins can cause mitochondrial outer membrane permeabilization and cytochrome c release as the trigger for intrinsic apoptosis induction. The Bcl-2 family comprises both proapoptotic and antiapoptotic proteins, depending on their BH domain composition, and it is the balance between both types of proteins that determines whether the mitochondrial pathway to apoptosis is activated [80].

Regarding BIM, several SRSF1-induced transcript variants were described lacking exons 2, 3, or 4 (BIM *γ*1, *γ*2, ES) which encode the BH3 domain. This domain binds antiapoptotic Bcl-2 family members and is necessary for induction of apoptosis by BIM [79, 81]. Similarly, SRSF1 expression promotes inclusion of exon 2 of the BH3 domain-containing gene MCL-1 (myeloid cell leukemia-1) giving rise to the antiapoptotic MCL-1L isoform in both breast cancer and choriocarcinoma cells [82]. Overexpression of SRSF1 also promotes generation of the antiapoptotic isoform BCL-XL [83].

BIN1 has tumor-suppressor activity by interacting with and activating MYC-mediated apoptosis, except when exon 12A is included by SRSF1-mediated alternative splicing, because the resulting antiapoptotic BIN1+12A isoform is unable to interact with MYC. Furthermore, SRSF1 was shown to modulate exclusion of exon 5 of the mRNA encoding the inhibitor of caspase-activated DNase (ICAD), a regulator of the DNase responsible for DNA fragmentation during apoptosis [84].

A parallel group of SRSF1-regulated target genes is involved in cellular signaling pathways related to proliferation and cell cycle progression. Examples of genes from this group are* CCND1*,* RPS6KB1*,* RON*,* RAC1,* and* MKNK2* genes.

SRSF1 increases expression of the cyclin D1b oncogene which arises from alternative splicing of the* CCND1* transcript, and harbors enhanced oncogenic functions not shared by full-length cyclin D1 (cyclin D1a) [85]. In this case, SRSF1 blocks recognition of the* CCND1* exon 4-intron 4 boundary, thus repressing inclusion of exon 5 so that a nuclear protein with a unique C-terminus is generated. SRSF1 also promotes the inclusion of exon 5 into the pre-mRNAs of* TEAD-1* (TEF-1 or TCF13), a transcription factor normally involved in cell differentiation and cell cycle arrest in myoblasts [86].

The* RPS6KB1* gene encodes the protein S6 kinase 1, a substrate for the cell growth regulating kinase mTOR. Excess SRSF1 promoted an increase in S6K1 variants by including one to three alternative cassette exons between exons 6 and 7 that are normally skipped [73] and include a proper stop codon. These short S6K1 isoforms have a truncated kinase domain and lack the mTOR-regulated C-terminus but are able to bind to and activate the mTORC1 complex. This activation of the mTORC1 complex occurs independent of the classical PI3K/AKT pathway and leads to phosphorylation of eIF4EBP1, releasing its inhibitory effect on cap-dependent translation [87, 88]. In addition, there is evidence that SRSF1 itself participates in a complex with mTORC1 to enhance translation efficiency of its target transcripts [63], for example, survivin [89] and *β*-catenin [90].

*RON* encodes a receptor tyrosine kinase in breast and colon tumors and SRSF1 promotes skipping of exon 11 by binding to an enhancer element in the competing exon 12. The resulting isoform ΔRon is constitutively active and promotes cell motility [91] as part of an epithelial-mesenchymal transition program, which tumor cells may use to escape from adverse local growth conditions.

Breast and colon tumors are further characterized by overexpression of Rac1b [92, 93], a hyperactivated splice variant of the small GTPase Rac1, which is involved in gene transcription and cell motility [94, 95]. In colorectal cells, SRSF1 was shown to be required for inclusion of an additional exon 3b to generate Rac1b [96] and increased expression of Rac1b contributes to cell survival [97, 98].

Finally, SRSF1 overexpression enhances the inclusion of the alternative 3′-terminal exon 13b of the gene* MKNK2* encoding the protein kinase Mnk2, an effector in the ERK/MAPK pathway [73]. The corresponding isoform Mnk2b lacks a C-terminal MAPK-binding domain and does not phosphorylate and activate the p38-MAPK required for stress-induced cell death. In contrast, it sustains phosphorylation of the translation initiation factor eIF4E, thus promoting cap-dependent protein translation and cell growth [99].

Curiously, splicing of the Mnk2b isoform was not induced when a chimeric nucleus-retained SRSF1 protein (SRSF1-NRS1), fused to the nuclear retention signal of the nonshuttling protein SRSF2, was overexpressed, suggesting an indirect effect of SRSF1 for this splicing event. Nevertheless, SRSF1-NRS1 was as competent as wild-type SRSF1 in inducing mammary cell transformation in 3D cultures but requires presence of the RRM1 domain, revealing a significant contribution of the nuclear functions of SRSF1 to cell transformation [79].

In hepatocellular xenografts, however, SRSF1-NRS1 protein had a much lower effect on tumor formation than SRSF1. In this model, SRSF1 overexpression also promotes activation of ERK/MAPK, probably by increasing B-RAF mRNA and protein levels. Although the mechanism remains to be explained, the RRM1 domain is required and sufficient to induce activation of this oncogenic pathway, indicating that the effect occurs at the mRNA level [81]. These findings reveal that SRSF1 can exert its oncogenic role through both nuclear and cytosolic pathways depending on the cellular contexts.

More recently, SRSF1 overexpression was also reported in lung cancer and novel SRSF1 target transcripts were identified, including the genes, ATP11C, IQCB1, TUBD1, proline-rich coiled-coil 2C (PRRC2C) [100], and survivin [89].

Apart from the genetic changes affecting proliferation and survival of the transformed cancer cells themselves, another important aspect of cancer cell biology is their cellular communication with the surrounding stroma. It is imperative for the growth of epithelial tumors to gain access to nutrient supply via blood vessels so that cancer cells release angiogenic signals to endothelial cells, for instance, the vascular endothelial growth factor VEGF. SRSF1 is involved in promoting proximal splice site selection in C-terminal exon 8 of VEGF, resulting in the generation of proangiogenic isoforms [101, 102]. Besides overexpression, this can result from SRSF1 activation in epithelial cells following oncogenic signaling through IGF-1, EGF, or TNF-*α*. These factors lead to activation of SRPKs [26, 42], which then phosphorylate SR proteins including SRSF1. SRPK1 inhibition has been used to manipulate the local balance of pro- and antiangiogenic in eye pathologies caused by neovascularization [103] and might be interesting for cancer therapy. In addition, SRSF1-mediated alternative splicing of both Ron and TEAD-1 has been linked to increased expression of angiogenic growth factors [77, 104]. SRSF1 may thus impact VEGF expression through both direct and indirect regulation to promote angiogenesis.

The role of fibroblasts in the stroma is to deposit or remodel extracellular matrix components and this is important for tumor cell migration. For example, a dense fibronectin meshwork favors epithelial cell invasion and results from inclusion of the EDA exon through SRSF1-regulated alternative splicing of the unique fibronectin-encoding gene [105, 106]. This occurs during embryogenesis but also in adult fibroblasts during tissue repair, tumor progression, and inflammation when expression levels of SRSF1 increase. It remains to be established whether tumor cells can release signals that induce increased SRSF1 expression in tumor-associated fibroblasts.

Following therapeutic challenge of tumor cells with DNA-damaging agents, resistant cells can eventually emerge. In one report hyperphosphorylation of SRSF1 was observed in the presence of DNA damage, causing altered subnuclear distribution and changes in alternative splicing pattern of target genes that promote cell survival [49]. Similarly, treatment of pancreatic tumor cells with the nucleoside analogue gemcitabine induced SRSF1 overexpression, and the resulting splicing of MNK2b with consequent phosphorylation of the translation initiation factor eIF4E was identified as the cause for drug resistance [107]. Furthermore, non-small cell lung cancer (NSCLC) cells respond to daunorubicin or cisplatin with an antiapoptotic caspase 9b splice variant. SRSF1 regulates this alternative splicing event by binding to a splicing enhancer in intron 6 and subsequent exclusion of an exon 3,4,5,6-cassette, generating caspase 9b [108]. SRSF1 is activated following hyperphosphorylation at serines 199, 201, 227, and 234 [109], mediating the therapeutic resistance of NSCLC. Another study in NSCLC observed that SRSF1 protein accumulates when cells were treated with carboplatin and paclitaxel and that cells stably overexpressing SRSF1 were more resistant to these chemotherapeutic drugs [74].

## 9. Conclusions

SRSF1 is an important protein for the regulation of constitutive and alternative splicing of cellular pre-mRNAs. Its activity as splicing regulator depends on the relative expression level of SRSF1 compared to other antagonistic or synergistic splicing factors as well as on its posttranslational modifications. In particular, the phosphorylation state of SRSF1 determines its nuclear or cytoplasmic localization and proteolytic degradation. Overexpression of SRSF1 has been reported in various tumors types and this has consequences for the alternative splicing profile expressed in tumor cells. Clear experimental evidence for tumor-promoting effects of SRSF1-induced alternative splicing variants has been provided but the genome-wide scale of its effects on cancer cell biology remains to be described.

Similar studies on other splicing factors are beginning to emerge and will likely reveal comparable complex effects on cancer cell transcriptomes as part of an adaptive response to activate survival pathways in tumor cells. A more comprehensive knowledge of these pathways may allow designing therapeutic interventions based on a combination of inhibitory drugs targeting simultaneously various pathways to reduce the selection of therapy-resistant tumor cell clones.
